# DNA methylation is associated with downregulation of the organic cation transporter OCT1 (SLC22A1) in human hepatocellular carcinoma

**DOI:** 10.1186/gm298

**Published:** 2011-12-23

**Authors:** Elke Schaeffeler, Claus Hellerbrand, Anne T Nies, Stefan Winter, Stephan Kruck, Ute Hofmann, Heiko van der Kuip, Ulrich M Zanger, Hermann Koepsell, Matthias Schwab

**Affiliations:** 1 Dr Margarete Fischer-Bosch Institute of Clinical Pharmacology, Auerbachstrasse 112, 70376 Stuttgart, Germany; 2 Department of Internal Medicine I, University Hospital Regensburg, Franz-Josef-Strauss-Allee 11, 93053 Regensburg, Germany; 3 Department of Urology, University Hospital Tuebingen, Hoppe-Seyler-Strasse 3, 72076 Tuebingen, Germany; 4 Institute for Anatomy and Cell Biology, University of Wuerzburg, Koellikerstrasse 6, 97070 Wuerzburg, Germany; 5 Department of Clinical Pharmacology, University Hospital Tuebingen, Otfried-Mueller-Strasse 45, 72076 Tuebingen Germany

## Abstract

**Background:**

Organic cation transporters (OCTs) determine not only physiological processes but are also involved in the cellular uptake of anticancer agents. Based on microarray analyses in hepatocellular carcinoma (HCC), *SLC22A1*/*OCT1 *mRNA seems to be downregulated, but systematic protein expression data are currently missing. Moreover, the underlying molecular mechanisms responsible for altered *SLC22A1 *expression in HCC are not fully understood. Therefore, we investigated the role of DNA methylation in the transcriptional regulation of the family members *SLC22A1*/*OCT1, SLC22A2*/*OCT2 *and *SLC22A3*/*OCT3 *in HCC.

**Methods:**

Semiquantitative immunohistochemistry of SLC22A1 protein expression was performed in paired HCC and histological normal adjacent liver tissues (*n *= 71) using tissue microarray analyses, and the results were correlated with clinicopathological features. DNA methylation, quantified by MALDI-TOF mass spectrometry and gene expression of *SLC22A1, SLC22A2 *and *SLC22A3 *were investigated using fresh-frozen HCC (*n *= 22) and non-tumor adjacent liver tissues as well as histologically normal liver samples (*n *= 120) from a large-scale liverbank.

**Results:**

Based on tissue microarray analyses, we observed a significant downregulation of SLC22A1 protein expression in HCC compared to normal adjacent tissue (*P *< 0.0001). SLC22A1 expression was significantly inverse correlated with expression of the proliferation marker MIB1/Ki-67 (r_s _= -0.464, *P *< 0.0001). DNA methylation of *SLC22A1 *was significantly higher in HCC compared with non-tumor adjacent liver tissue and was lowest in histologically normal liver tissue. Methylation levels for *SLC22A1 *in combination with *RASSF1A *resulted in a specificity of > 90% and a sensitivity of 82% for discriminating HCC and tumor-free liver tissue.

**Conclusions:**

DNA methylation of *SLC22A1 *is associated with downregulation of SLC22A1 in HCC and might be a new biomarker for HCC diagnosis and prognosis. Moreover, targeting *SLC22A1 *methylation by demethylating agents may offer a novel strategy for anticancer therapy of HCC.

## Background

Hepatocellular carcinoma (HCC) is the sixth most common cancer worldwide and the third most common cause of cancer-related death. Understanding of the molecular pathogenesis of HCC has improved, and some progress has been made in translating these findings into clinical practice [1]. Therapeutic options include sorafenib, as first effective systemic treatment against HCC, as well as radioembolization and oncolytic approaches. Currently, there are more than 150 ongoing clinical trials. But, in the main, these are not integrating information about the molecular pathogenesis of HCC [2]. For instance, the cellular uptake of anticancer drugs is an important first step in the mechanism of drug action. Therefore, multidrug resistance can result not only from increased efflux but also reduced uptake of drug into tumor cells. Uptake transport in hepatocytes is mediated by members of the solute carrier (SLC) family. Organic cation transporters (OCTs) are involved in the transport of a variety of endogenous and exogenous organic cationic compounds and thus the expression of these transporters is suggested to be a significant determinant of physiological functions in different organs [3-5]. OCTs are also important drug targets since several clinically relevant agents, including anticancer drugs (for example, platinum agents), are substrates of OCTs.

Expression profiles among the three OCT family members OCT1 (encoded by *SLC22A1*), OCT2 (*SLC22A2*) and OCT3 (*SLC22A3*) are extremely diverse in different tissues [6]. *SLC22A1 *is expressed mainly in normal human liver, and *SLC22A2 *is predominately expressed in kidney [3-5,7,8]. There is already evidence that OCTs are differentially expressed in tumor tissues, and based on microarray data, *SLC22A1 *mRNA expression is downregulated in HCC [9-11]. However, systematic analyses of expression of *SLC22A1, SLC22A2 *and *SLC22A3 *have not been performed so far in HCC. Moreover, the underlying mechanisms responsible for altered expression of *SLC22A1 *in HCC compared with normal liver are poorly understood.

Epigenetic alterations, including DNA methylation and histone modifications, are important mechanisms in tumorigenesis. DNA hypermethylation-induced transcriptional silencing of tumor suppressor and DNA repair genes is a frequent phenomenon [12]. There is already proof of principle for the clinical value of methylation markers for classification (for example, colorectal cancer [13,14]), prognosis [15] and prediction of therapeutic response [12]. Thus, identification of specific expression-regulating core gene regions (that is, CpG dinucleotides) in the promoter is essential. With respect to OCTs, there is evidence that kidney-specific expression of *SLC22A2 *is regulated by DNA methylation [16]. Currently there are no data on whether downregulation of SLC22A1 in HCC could be explained by hypermethylation of the *SLC22A1 *promoter. Elucidating the precise mechanism for downregulation of SLC22A1 in HCC is especially important because it is possible to overcome gene silencing with demethylating agents like decitabine, which opens new therapeutic strategies for HCC.

Therefore, in the present study we performed systematic immunohistochemical analysis of SLC22A1 protein in well-characterized, paired HCC and corresponding non-tumor tissues and correlated the results with clinicopathological data. We elucidated systematically the influence of DNA methylation on the transcriptional regulation of *SLC22A1, SLC22A2 *and *SLC22A3 *in HCC tissues and in tissues in a large human liver bank.

## Materials and methods

### Tumor and non-neoplastic human tissues

An overview about different sample sets used in the present study is given in Table S1 in Additional file 1. HCC tissues and corresponding adjacent non-tumor liver tissues from patients who underwent surgical resection between 2001 and 2007 were collected at the Department of Internal Medicine I, University Hospital Regensburg, Regensburg, Germany and used for tissue microarray analysis (TMA), as previously described [17,18]. Detailed clinicopathological characteristics of patients (*n *= 71) are given in Table S2 in Additional file 1. Additionally, paired fresh-frozen HCC tissues (*n *= 15) and adjacent non-tumor liver samples were obtained from the same institution [19]. As additional samples, seven HCCs were obtained from OriGene (OriGene, Rockville, MD, USA) and re-examined by a pathologist. The patient characteristics are summarized in Table S3 in Additional file 1. Finally, as a control group, 120 histologically normal fresh-frozen liver tissues (IKP-liverbank; Table S4 in Additional file 1) were investigated, which were collected at the Department of General, Visceral and Transplantation Surgery (Campus Virchow, University Medical Center Charité, Humboldt University Berlin, Germany) as previously reported [20]. For systematic expression profiling of *SLC22A1, SLC22A2 *and *SLC22A3 *in normal and tumor tissues, commercial arrays including cDNA from 381 human tissues normalized to beta-actin were used (TissueScan, Origene technologies, Rockville, MD, USA). The study conforms to the ethical guidelines of the 1975 Declaration of Helsinki and was approved by the ethics committee of the University Hospital Regensburg (Regensburg, Germany), by the ethics committee of University of Tuebingen (Tuebingen, Germany) and the Charite (Berlin, Germany). Written informed consent was provided by each patient.

### RNA isolation and TaqMan analysis

Total RNA was extracted from HCCs and non-tumor tissue using the mirVana™ miRNA Isolation Kit (Applied Biosystems/Ambion, Austin, TX, USA). High quality liver RNA was extracted as previously described [20], and only high-quality RNA preparations with RNA Integrity Number (RIN) assignment (> 7), as determined by Agilent Bioanalyzer (Nano LabChip Kit, Agilent Technologies, Waldbronn, Germany), were used. Total RNA was used for cDNA syntheses using the High Capacity cDNA Reverse Transcription Kit (Applied Biosystems, Foster City, CA, USA). mRNA expression was quantified by TaqMan technology (for details see additional methods in Additional file 1).

### Immunofluorescence microscopy of tissue samples

Localization of OCTs in liver and HCC tissues was analyzed by immunofluorescence confocal laser scanning microscopy (for details see additional methods in Additional file 1).

### HCC tissue microarray analysis

TMAs were processed as previously described [17,18]. SLC22A1 immunohistochemistry was performed using a polyclonal antiserum raised in rabbits against a synthetic peptide corresponding to the carboxy-terminal sequence of human SLC22A1 as described previously [20]. Staining of SLC22A1 was performed according to the following protocol: 5 μm TMA sections were transferred to slides (Superfrost-Plus, Langenbrinck, Teningen, Germany). Tissues were deparaffinized by passing the specimens through xylene and rehydrated through serial dilutions of ethanol (100%, 96% and 70%). Heat-induced antigen retrieval was accomplished using a solution buffer at pH 6.0 (Dako Cytomation, Glostrup, Sweden) for 30 minutes. Endogenous peroxidase was blocked with a peroxidase blocker (0.03% H_2_O_2_; Dako Cytomation) for 10 minutes. Slides were incubated for 30 minutes at room temperature with the primary antibody (OCT1/KEN, dilution 1:2,000) in an antibody diluent (Dako Cytomation). For negative control the primary antibody was omitted. The REAL-HRP system (REAL Envision Detection System, Rabbit/Mouse, Dako Cytomation) was used for detection of the antibody binding before sections were counterstained with hematoxylin and mounted. For semiquantitative immunohistochemical assessment first, staining intensity was graded according to the following scoring system: 0 = negative, 1 = low, 2 = medium and 3 = high. As previously described [21,22], the final immunoreactivity score was calculated by multiplication of the staining intensity score (0 to 3) and the percentage of immunoreactive (stained) cells (0 to 100%), resulting in a numeric value of 0 to 300. MIB1 was analyzed applying the anti-Ki-67 antibody (clone MIB1, 1:10, final concentration of 5 μg/ml; DAKO) to assess the MIB1/Ki-67 proliferation rate as previously described [17]. Evaluation of the staining was done by two investigators blinded to the patient characteristics.

### DNA methylation analysis

Quantitative DNA methylation analysis was performed with matrix-assisted laser desorption ionization time-of-flight mass spectrometry (MALDI-TOF MS; Sequenom, San Diego, CA, USA), as described in the additional methods in Additional file 1. Mass spectra were acquired with the MassArray Compact MALDI-TOF MS (Sequenom) and spectra methylation ratios were analyzed using the Epityper 1.0 software (Sequenom).

### Statistical methods

Statistical analyses were performed with R-2.13.0, PASW Statistics 17.0.2 and GraphPadPrism 4.03 (GraphPad Software Inc., La Jolla, CA, USA). Statistical significance was defined as *P *< 0.05. For a detailed description, see the additional methods in Additional file 1.

## Results

### Expression profiling of *SLC22A1, SLC22A2*, and *SLC22A3 *in normal and tumor tissue

**Figure 1:**
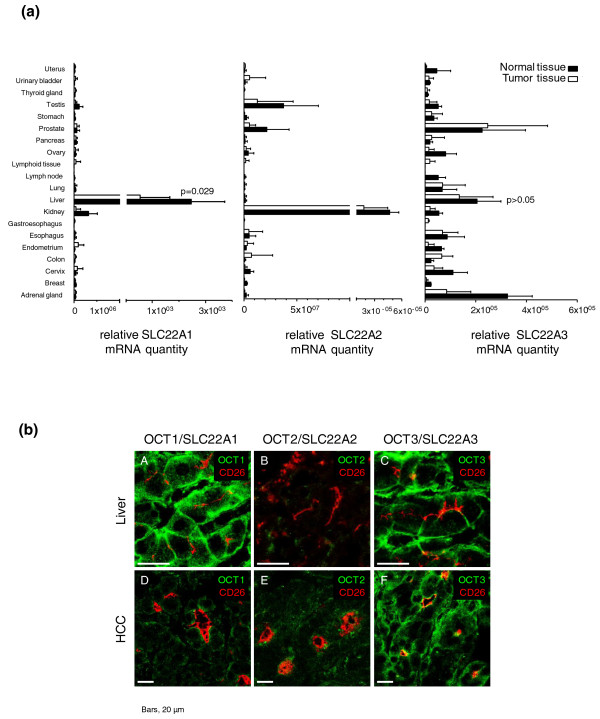
**Expression analysis in normal and tumor tissues**. **(a) ***SLC22A1, SLC22A2*, and *SLC22A3 *mRNA expression in different human normal and tumor tissues of the respective organs (TissueScan, Origene Technologies, Rockville, MD, USA) was quantified using TaqMan technology. **(b) **SLC22A1/OCT1, SLC22A2/OCT2 and SLC22A3/OCT3 protein expression (green) was analyzed by confocal laser scanning microscopy in HCC tumor tissue (*n *= 7) compared with histologically normal liver tissue (*n *= 5, IKP-liverbank) derived from patients without hepatocellular carcinoma (HCC). Specific antibodies were used to detect SLC22A1, SLC22A2 and SLC22A3 proteins. Representative images are shown.

A comprehensive analysis using cDNAs from 381 human tissue samples (Table S1 in Additional file 1) from 20 different tissues revealed that *SLC22A1 *mRNA is expressed most prominently in normal liver and expressed at significantly lower levels in HCC tissues (*P *= 0.029; Figure 1A). *SLC22A2 *transcripts were present in human kidney, but almost absent in human normal liver, as well as in HCC tissue. *SLC22A3 *transcripts were detectable in a variety of human tissues, including human liver (Figure 1a), and *SLC22A3 *expression did not differ significantly between normal and tumor liver tissue.

Moreover, specific antibodies were used to detect SLC22A1, SLC22A2 and SLC22A3 protein by immunofluoresence confocal laser scanning microscopy in HCC tumor tissue (*n *= 7 from HCC cohort 1; Tables S1 and S3 in Additional file 1) and histologically normal liver tissue (*n *= 5 from IKP-liverbank; Tables S1 and S4 in Additional file 1) derived from patients without HCC. In line with previous data, only SLC22A1 and SLC22A3 were detected in human liver [20]. In HCC tissue, SLC22A3 and, at a lower level, SLC22A1 proteins were detected. SLC22A2 was not detected in either normal or HCC tissue (Figure 1b).

### Protein expression analysis of SLC22A1 in HCC

**Figure 2:**
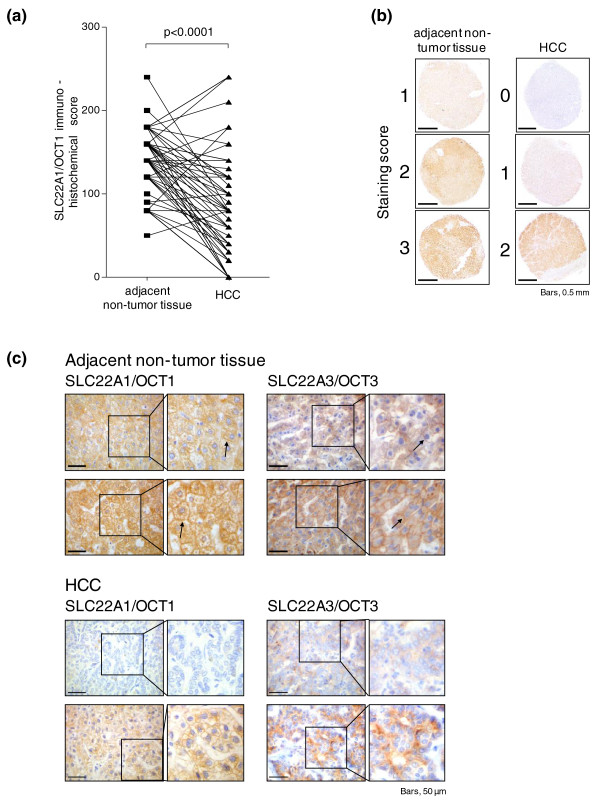
**Immunohistochemical staining of SLC22A1 protein in hepatocellular carcinoma and histological non-tumor adjacent tissue**. **(a) **Expression of SLC22A1 is significantly decreased in hepatocellular carcinoma (HCC) versus adjacent non-tumor tissue (*P *< 0.0001) and individual paired results are given. For each patient, score values from both tissues are connected by a line. **(b) **Examples of different staining intensities (0 = negative, 1 = low, 2 = medium, 3 = high) in non-tumor as well as HCC tissues. SLC22A1 was present in all non-tumor tissues. **(c) **Exemplary weak immunohistochemical staining of SLC22A1 in two HCC samples is shown compared with strong staining in adjacent non-tumor liver tissue. In addition, representative examples of SLC22A3 staining in HCC tissue as well as in adjacent non-tumor liver tissue is shown. SLC22A1 and SLC22A3 are detected in the sinusoidal membrane of the hepatocytes (black arrows).

To systematically evaluate expression of SLC22A1 protein in HCC tissues, levels were analyzed by semi-quantitative immunohistochemistry using TMA of HCC and corresponding adjacent non-tumor tissues [17,18]. Investigation of SLC22A1 protein expression was informative in 68 HCC and 67 adjacent tissues of this HCC study population (set 1; for details see Tables S1 and S2 in Additional file 1), because some of the cores on TMAs were lost or damaged during processing of the TMAs. The expression of SLC22A1 was determined by applying an immunohistochemical score, as described in the Materials and methods. Statistical analysis revealed significantly lower SLC22A1 immunoreactivity in HCC tissues compared with adjacent tissues (*P *< 0.0001; Figure 2a), irrespective of the presumed etiology of the HCC (for example, hepatitis B virus or hepatitis C virus; a paired comparison is shown in Figure S1 in Additional file 1). Examples of different staining intensities (0 = negative, 1 = low, 2 = medium, 3 = high) in non-tumor as well as in HCC tissue are shown in Figure 2b. SLC22A1 expression in HCC tissues, when present, was variable, ranging from at least 20 to 90% of the cells, and showed a more diffuse pattern compared with non-tumor tissue (Figure 2c). In addition, expression of SLC22A3 was analyzed for a subset of HCC samples, used for immunostaining of SLC22A1. As shown in Figure 2c, immunostaining in these samples clearly demonstrates SLC22A3 expression at the protein level.

When we correlated the SLC22A1 immunoreactivity score with clinicopathological characteristics, a significant inverse correlation was found only for the MIB1/Ki-67 proliferation rate (r_s _= -0.464, *P *< 0.0001), which was determined in HCC tissue samples by immunohistochemical staining as previously described (Figure S2A in Additional file 1) [18]. Moreover, a significant difference in SLC22A1 expression between samples with low (≤5%) and high (> 5%) proliferation rate was found (*P *= 0.01; Figure S2B in Additional file 1). In contrast, no association was detected with etiological factors (for example, hepatitis B virus or hepatitis C virus), age at surgery or sex. Moreover, tumor grade or histological stage was not significantly associated with SLC22A1 expression, although both stage 4 tumors revealed the lowest levels of protein expression (Figure S3A,B in Additional file 1).

### DNA methylation of *SLC22A1, SLC22A2 *and *SLC22A3 *in HCC and adjacent non-tumor tissues

**Figure 3:**
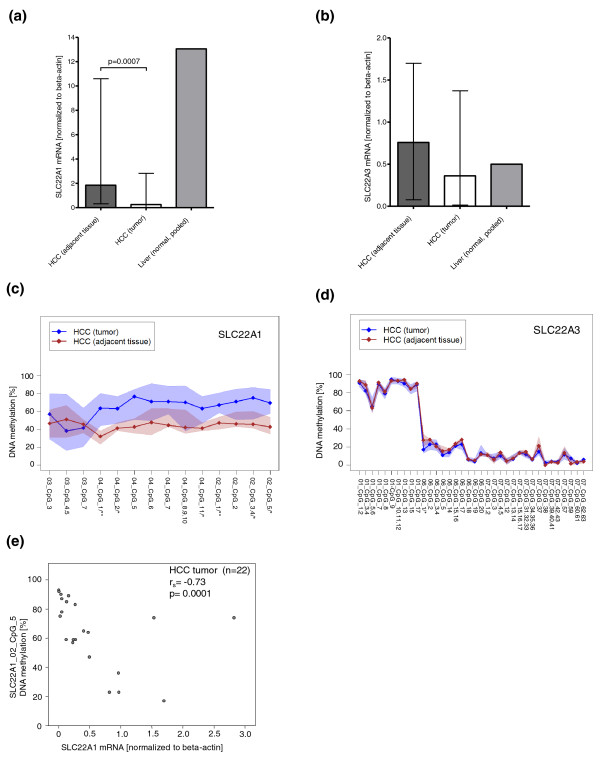
**Expression and DNA methylation of *SLC22A1 *and *SLC22A3 *in hepatocellular carcinoma and adjacent non-tumor tissue**. **(a,b) **Quantitative mRNA expression of *SLC22A1 *(a) and *SLC22A3 *(b) in hepatocellular carcinoma (HCC), adjacent non-tumor tissue and histologically normal liver tissue (pooled *n *= 100, IKP-liverbank). Data represent median and range. **(c,d) **DNA methylation profiles of *SLC22A1 *(c) and *SLC22A3 *(d) in HCC and adjacent non-tumor tissue. DNA methylation (y-axis) at each individual CpG site (x-axis) is given. Methylation profiles show median levels (diamonds) at each CpG position (shaded areas are defined by 25%/75% quantiles). *P*-values are given for differences in methylation level for CpG sites. *P < 0.05, **P < 0.01 are given on the x-axis. **(e) **Correlation of *SLC22A1 *mRNA expression in the 22 HCC samples with DNA methylation of one individual CpG site (SLC22A1_02_CpG_5) in *SLC22A1*.

To confirm the finding of a significantly lower expression of SLC22A1 in HCC, mRNA levels of *SLC22A1, SLC22A2*, and *SLC22A3 *were determined by real-time PCR analysis in a cohort of 22 fresh-frozen HCC tissues and adjacent non-tumor liver samples (HCC study population set 2; Tables S1 and S3 in Additional file 1). Moreover, expression levels were compared with histologically normal liver tissue from patients without primary liver tumors (*n *= 100 pooled cDNAs, IKP-liver bank; Tables S1 and S4 in Additional file 1). As shown in Figure 3a, *SLC22A1 *mRNA expression in HCC samples was significantly lower (*P *= 0.0007) compared with adjacent tissue. Generally, *SLC22A3 *mRNA did not differ significantly between HCC and adjacent tissue (Figure 3b). Again, the *SLC22A2 *mRNA levels in HCC and adjacent tissue was very low (data not shown).

To evaluate whether SLC22A1 is silenced in HCC due to hypermethylation of *SLC22A1 *in tumor tissue, we studied tissue-specific DNA methylation. DNA methylation of *SLC22A1, SLC22A2*, and *SLC22A3 *gene promoters was studied by gene-specific amplification of bisulfite-treated DNA followed by *in vitro *transcription and MALDI-TOF MS analysis [23,24]. The amplification regions were designed to cover the surrounding 5' UTRs of the target genes. In addition, promoter regions were analyzed *in silico *for the presence of CpG islands. In contrast to *SLC22A2 *and *SLC22A3*, no CpG island was predicted in the promoter region of *SLC22A1*, which is supported by genome-wide CpG island mapping analysis [25]. We were able to quantify 18 CpG sites for *SLC22A1*, 6 sites for *SLC22A2 *and 57 sites for *SLC22A3 *(Table S5 in Additional file 1). As described in detail in the additional methods in Additional file 1 the MALDI-TOF MS method does not always allow detection of methylation levels for each single CpG site [24], and some of the methylation values represent the methylation state of subsequent CpG sites. As shown in Figure 3c, *SLC22A1 *methylation was significantly increased in HCC tissues, consistent with the low level of mRNA expression. For both HCC and adjacent non-tumor liver tissue, DNA methylation of *SLC22A1 *was highly variable between individuals. In contrast, *SLC22A3 *methylation was generally not different between HCC tissues and adjacent non-tumor liver tissue (Figure 3d). DNA methylation of *SLC22A2 *in HCC and adjacent non-tumor liver tissue was high and showed low inter-individual variability (Figure S4A in Additional file 1).

When we correlated DNA methylation with *SLC22A1 *mRNA expression in the total group of 22 HCC samples (HCC study population, set 2; Tables S1 and S3 in Additional file 1), a significant inverse correlation between *SLC22A1 *mRNA expression and individual CpG sites within the *SLC22A1 *promoter region was found (Figure 3e; Table 1). There was no correlation between *SLC22A1 *mRNA expression and DNA methylation with clinicopathological variables like age at surgery, sex, tumor grade and histological stage and tumor size (Figure S5A-D in Additional file 1).

**Table 1:** Correlation analysis of DNA methylation of individual CpG sites and *SLC22A1 *mRNA expression in hepatocellular carcinoma

CpG sites	Spearman's correlation coefficient	*P*-value	Holm-adjusted *P*-value
SLC22A1_03_CpG_3	-0.274	0.217	0.652
SLC22A1_03_CpG_4_5	-0.221	0.323	0.652
SLC22A1_03_CpG_7	-0.425	0.048	0.247
SLC22A1_04_CpG_1	-0.525	0.012	0.110
SLC22A1_04_CpG_2	-0.451	0.035	0.247
SLC22A1_04_CpG_5	-0.381	0.080	0.321
SLC22A1_04_CpG_6	-0.129	0.568	0.652
SLC22A1_04_CpG_7	-0.590	0.004	0.039
SLC22A1_04_CpG_8.9.10	-0.462	0.030	0.242
SLC22A1_04_CpG_11	-0.451	0.035	0.247
SLC22A1_02_CpG_1	-0.709	0.0002	0.003
SLC22A1_02_CpG_2	-0.601	0.003	0.034
SLC22A1_02_CpG_3_4	-0.620	0.002	0.025
SLC22A1_02_CpG_5	-0.726	0.0001	0.002

*P*-values correspond to two-sided Spearman correlation tests.

### DNA methylation of *SLC22A1, SLC22A2*, and *SLC22A3 *in histologically normal liver tissue

**Figure 4:**
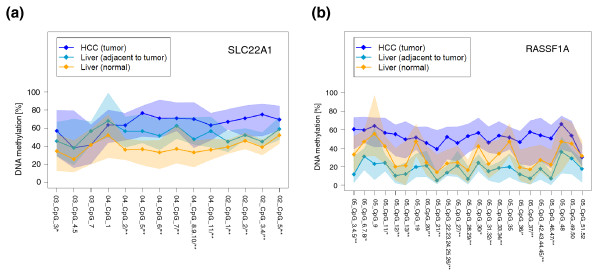
**DNA methylation of *SLC22A1 *and *RASSF1A *in histologically normal liver tissue**. **(a,b) **DNA methylation profiles of *SLC22A1 *(a) and *RASSF1A *(b) in normal liver tissue (*n *= 100, IKP-liverbank), in histologically normal tissues derived from patients with hepatocellular carcinoma (HCC; *n *= 20, adjacent to tumor) and in HCC tumor tissue (*n *= 22). DNA methylation (y-axis) at each individual CpG site (x-axis) is given. Methylation profiles show median levels (diamonds) at each CpG position (shaded areas are defined by 25%/75% quantiles). *P*-values are given for differences in methylation level for CpG sites between HCC (*n *= 22) and histologically normal liver tissue (*n *= 100). *P < 0.05, **P < 0.01.

Generally, aberrant DNA methylation has been demonstrated in tumor tissue and adjacent, histologically normal tissue of tumor patients [26]. We therefore examined *SLC22A1, SLC22A2 *and *SLC22A3 *DNA methylation in histologically normal liver tissue from patients without primary liver tumors (*n *= 100, IKP-liverbank; Tables S1 and S4 in Additional file 1) compared with histologically normal tissues derived from patients with HCC (*n *= 20; Tables S1 and S4 in Additional file 1). Significantly higher levels of methylation of *SLC22A1 *(Figures 4A and S6A in Additional file 1), but not *SLC22A3 *(Figure S6C in Additional file 1) were observed in adjacent tissue from primary liver tumors (*n *= 20) compared with normal livers without primary liver tumors (*n *= 100). The *SLC22A2 *gene region was again highly methylated (Figure S6B in Additional file 1).

In summary, *SLC22A1 *DNA methylation was highest in HCC tissue (HCC study population set 2) and decreased progressively from adjacent to histologically normal tissue of patients without primary liver tumors from the IKP-liverbank (Figure 4a). Since a similar behavior has been reported for the tumor suppressor gene *RASSF1A *[27-29], DNA methylation levels of *RASSF1A *were determined in our study. As indicated in Figure 4b, we confirmed DNA hypermethylation of the tumor suppressor gene *RASSF1A *in HCC samples of our study. Comparable to our data on *SLC22A1 *methylation, DNA methylation levels of *RASSF1A *were higher in HCC tissue compared with adjacent non-tumor tissue (Figure 4b). However, in contrast to *SLC22A1 *(Figure 4a), methylation of *RASSF1A *was not lower in histologically normal tissue of patients without primary liver tumors (*n *= 100) compared with normal tissues derived from patients with HCC (*n *= 20, IKP-liverbank; Figure 4b).

To investigate whether DNA methylation status of *SLC22A1 *and *RASSF1A *could effectively distinguish HCC and histologically normal tissue, we determined sensitivity and specificity (see statistical methods in Additional file 1). The methylation levels of 11 selected CpG sites in *SLC22A1 *and two in *RASSF1A *together could classify HCC and normal tissues with 82% sensitivity and 96% specificity. For CpG sites of *RASSF1A *alone, specificity was only 86% in contrast to 96% for CpG sites of *SLC22A1*. The sensitivity was similar (64%) for both genes.

## Discussion

Altered expression of OCT transporters is important for physiological processes and therapeutic responses to drugs. Indeed, many clinically important drugs (for example, metformin, cisplatin) are substrates for OCTs [3-5,30]. Independent of genetic reasons for inter-individual differences in SLC22A1 and SLC22A3 expression in histologically normal liver tissue [20], there is first evidence from microarray studies that *SLC22A1 *mRNA expression is decreased in HCC [9-11]. This is supported by our experiments using cDNAs from 381 human tissue samples from 20 different tissues (Table S1 in Additional file 1). *SLC22A1 *mRNA is expressed most prominently in liver and at significantly lower levels in HCC tissues (*P *= 0.029; Figure 1a), which corresponds with weaker immunofluoresence staining by confocal laser scanning microscopy. There are studies to suggest that *SLC22A1 *is one of the most downregulated genes in HCC cells, with a gene pattern correlated to poor prognosis [10,31,32]. SLC22A1 also is expressed differentially in EpCAM positive, stem-cell-like-HCC and EpCAM negative mature hepatocyte-like HCC [33]. However, SLC22A1 protein expression in HCC has so far not been studied systematically and the molecular mechanisms underlying the tumor-specific expression of SLC22A1 are poorly understood.

Therefore, we investigated first protein expression in a set of 71 HCC tissues and adjacent non-tumor liver tissues by semiquantiative immunohistochemistry. These experiments indicated significantly decreased expression of SLC22A1 protein in HCC (Figure 2a). When we correlated SLC22A1 expression with clinicopathological characteristics, the most striking result was a significant inverse correlation between expression of SLC22A1 protein and the tumor proliferation rate MIB1/Ki-67. Previous findings support our observation since MIB1/Ki-67 staining is reduced in protein kinase JNK^-/- ^knockout mice [34] and microarray expression profiling clearly indicated that HCC tissues with high activation status of JNK1 showed reduced *SLC22A1 *mRNA expression and poorer prognosis [10].

Since altered DNA methylation is a common mechanism in tumor development, downregulation of SLC22A1 might be caused by hypermethylation of the *SLC22A1 *promoter region. This prompted us to systematically quantify DNA methylation levels of *SLC22A1, SLC22A2*, and *SLC22A3 *in the surrounding 5' UTRs of the target genes by MALDI-TOF MS. Consistent with both low expression of mRNA and protein, *SLC22A1 *methylation was significantly increased in HCC tissues compared with adjacent non-tumor (Figure 3c) or non-tumor liver tissues derived from patients without primary liver tumors (Figure 4a). Methylation of *SLC22A3 *was generally not different.

Of note, altered methylation has been reported not only for tumor tissue, but also for histologically normal tissue adjacent to the tumor, and is supposed to be an early and ubiquitous event in cancer development. Interestingly, we found significantly higher levels of *SLC22A1 *methylation (Figure 4a) but not *SLC22A3 *methylation in normal liver tissue adjacent to HCC tumors. This important finding may explain the discrepancy between our data and the results of Aoki *et al*. [16], who reported high methylation of *SLC22A1 *in a collection of 11 liver samples. Since Aoki *et al*. used only tumor-free tissue adjacent to HCC, these data strongly support our observation that *SLC22A1 *is hypermethylated in HCC and to a lesser extent in the adjacent, histologically tumor-free tissue.

Thus, based on our results, methylation of *SLC22A1 *decreased from high levels in HCC to lower levels in adjacent, histologically normal liver and was lowest in normal tissue from livers that were not derived from patients with HCC (Figure 4a). The same methylation pattern has been reported already for some tumor suppressor genes like *RASSF1A *[27]. *RASSF1A *was the best discriminator between HCC and tumor-free liver tissue [35,36], which is confirmed by our data showing highest methylation levels of *RASSF1A *in HCC compared with adjacent tumor-free liver tissue. Since early detection of aberrant DNA methylation might be beneficial for diagnosis of HCC, we suggest that *SLC22A1 *methylation might be a new biomarker for the diagnosis of HCC or for estimating risk for HCC in following patients at risk. This idea is supported by the observation that the MIB1/Ki-67 proliferation index correlated inversely with SLC22A1 expression in our HCC cohort. Additional analysis of whether DNA methylation effectively discriminates between HCC and histologically normal liver tissue indicated that the combination of the methylation status of *SLC22A1 *and *RASSF1A *had a higher specificity and sensitivity for this compared with each of the two markers alone. Further studies will be needed to verify these data.

Several causes for aberrant DNA methylation in HCC have been discussed. A recent study demonstrated that the methylation state in specific genes in HCC is influenced by viral infection or alcohol intake, thereby promoting hepatocarcinogenesis [28]. Genome-wide methylation analysis of human colon cancer showed that cancer-related methylation changes occur predominately at sites that distinguish normal tissues [37]. Interestingly, the same region in the *SLC22A1 *gene that is differentially methylated in HCC tissue in our study showed tissue-specific differences in *SLC22A1 *DNA methylation [38].

Since there is emerging evidence on the importance of the location of CpG dinucleotide hypermethylation in relation to gene expression but also clinicopathologic characteristics of tumors, we next performed a systematic correlation analysis between *SLC22A1 *mRNA expression and quantitative DNA methylation of individual CpG sites within the *SLC22A1 *promoter region. Using the total set of 22 fresh-frozen HCC tissues (Table S2 in Additional file 1), the CpG sites in *SLC22A1 *with the strongest inverse correlation with mRNA expression in HCC (Table 1) were remarkably located upstream of the translational start site in exon 1. However, the overall methylation profile for the total *SLC22A1 *region investigated in our study was quite similar. The underlying mechanism for this phenomenon is unknown, but recent findings indicate that especially DNA methylation of the first exon correlates with transcriptional silencing [39]. Thus, DNA methylation within the first exon may block effective transcription.

Since OCTs are important targets for various therapeutic agents, such as metformin or the platinum drugs cisplatin or picoplatin [30,40,41], the finding that *SLC22A1 *is downregulated in liver cancer due to hypermethylation may be important for alternative therapeutic approaches for treatment of HCC. We speculate that DNA methylation of *SLC22A1 *in human liver contributes to differences in drug response for drugs that are substrates of SLC22A1. By contrast with genetic variants, epigenetic modifications are reversible, which offers the possibility to modulate the expression of genes with demethylating agents (for example, decitabine). As a consequence, for instance, pretreatment with decitabine may increase the intracellular concentration of the SLC22A1 substrate cisplatin, thereby resulting in enhanced cytotoxicity. Of course, treatment with decitabine will not only lead to re-expression of OCTs and additional factors might be important for cisplatin efficacy, for example, the expression of the copper transporter CTR1, which alters sensitivity to cisplatin [42]. In addition, demethylation may impair expression of efflux transporters, thereby leading to an enhanced efflux and chemoresistance (reviewed in [43]). For instance, the promoter of the efflux transporter gene *ABCB1*, encoding P-glycoprotein, is hypomethylated in several cancer cell lines, thereby leading to a multidrug-resistance phenotype compared to drug-sensitive cell lines [44]. Moreover, the effect of decitabine treatment might lead to increased risk of cisplatin cytotoxicity in other organs, since the demethylating effect is not restricted to liver tissue.

Our study has some limitations that need to be considered. First, our study design of HCC samples was retrospective and, for instance, confounding of SLC22A1 expression cannot be excluded with certainty. Second, the number of HCC samples was small, and the relevance of the correlation analyses of distinct clinicopathological factors (for example, tumor stage, histological grade) with DNA methylation or expression is thus limited and needs further study. Nevertheless, our findings indicate for the first time that downregulation of the uptake transporter SLC22A1 in HCC is associated with DNA methylation as an epigenetic phenomenon. This finding has potential consequences for the diagnosis of HCC and for future therapeutic concepts.

## Conclusions

SLC22A1 protein expression is significantly decreased in HCC compared with tumor-free adjacent liver tissue. DNA hypermethylation of individual CpG sites within the *SLC22A1 *gene is associated with downregulation of SLC22A1 expression in HCC. Moreover, *SLC22A1 *methylation decreased from high levels in HCC to lower levels in histological tumor-free adjacent liver tissue and was lowest in tumor-free liver tissue not derived from patients with HCC. Therefore, early detection of aberrant DNA methylation of *SLC22A1 *might be beneficial for the diagnosis of HCC or for estimating risk for HCC in following patients at risk. Since platinum drugs are substrates of SLC22A1, the modulation of gene expression by pretreatment with demethylating agents may offer novel therapeutic options in HCC, which has to be proven in further studies.

## Abbreviations

HCC: hepatocellular carcinoma; MALDI-TOF MS: matrix-assisted laser desorption/ionization time-of-flight mass spectrometry; OCT: organic cation transporter; PCR: polymerase chain reaction; SLC: solute carrier; TMA: tissue microarray; UTR: untranslated region.

## Competing interests

The authors declare that they have no competing interests.

## Authors' contributions

MS was responsible for study design. ES was responsible for study design and collected, analyzed and interpreted data. Data were collected, analyzed and interpreted by ATN, CH and SK. Statistical analysis was performed by SW. All other authors contributed samples for the study, and/or contributed to drafting the manuscript. All authors have read and approved the manuscript for publication.

## Supplementary Material

Additional file 1 **Additional methods, tables and figures**. Methods: additional description of methods. Immunofluorescence microscopy of tissue samples was performed as previously described [20,45]. Primers used for DNA methylation analysis were designed with Methprimer software [46]. DNA methylation of *RASSF1A *was analyzed as described previously [47]. Statistical analyses were performed using the statistical software R, version 2.13.0 [48]. Where indicated, *P*-values were adjusted according to Holm's multiple testing correction procedure [49]. Table S1: overview of different sample sets used in the present study. Table S2: clinicopathological characteristics of 71 patients with HCC. Table S3: clinicopathological characteristics of HCC study population. Table S4: characteristics of histologically normal liver tissues (IKP-liverbank). Table S5: DNA methylation analysis of *OCT1/SLC22A1, OCT2/SLC22A2 *and *OCT3/SLC22A3*. Figure S1: differences in the SLC22A1 immunohistochemical score between the corresponding adjacent non-tumor tissue and HCC tissue, regarding the underlying etiology for HCC. Figure S2: Ki-67/MIB1 data for HCC tumor samples. Figure S3: association between SLC22A1 protein expression and tumor grade or tumor stage. Figure S4: DNA methylation profiles of *SLC22A2 *and *RASSF1A *in HCC and adjacent non-tumor tissue. Figure S5: association between *SLC22A1 *mRNA expression or DNA methylation and tumor stage or histological tumor grade. Figure S6: DNA methylation profiles of *SLC22A1, SLC22A2*, and *SLC22A3 *in non-tumor (normal) liver tissues, as well as in non-tumor liver tissue derived from patients with HCC. Click here for file
