# Supplementary material for: DNA methylation is associated with downregulation of the organic cation transporter OCT1 (SLC22A1) in human hepatocellular carcinoma
Source: Genome Med. 2011 Dec 23;3(12):82. doi: 10.1186/gm298 (PMC3334547; doi:10.1186/gm298)
Supplement: Additional file 1 — Additional methods, tables and figures. Methods: additional description of methods. Immunofluorescence microscopy of tissue samples was performed as previously described [20,45]. Primers used for DNA methylation analysis were designed with Methprimer software [46]. DNA methylation of RASSF1A was analyzed as described previously [47]. Statistical analyses were performed using the statistical software R, version 2.13.0 [48]. Where indicated, P-values were adjusted according to Holm's multiple testing correction procedure [49]. Table S1: overview of different sample sets used in the present study. Table S2: clinicopathological characteristics of 71 patients with HCC. Table S3: clinicopathological characteristics of HCC study population. Table S4: characteristics of histologically normal liver tissues (IKP-liverbank). Table S5: DNA methylation analysis of OCT1/SLC22A1, OCT2/SLC22A2 and OCT3/SLC22A3. Figure S1: differences in the SLC22A1 immunohistochemical score between the corresponding adjacent non-tumor tissue and HCC tissue, regarding the underlying etiology for HCC. Figure S2: Ki-67/MIB1 data for HCC tumor samples. Figure S3: association between SLC22A1 protein expression and tumor grade or tumor stage. Figure S4: DNA methylation profiles of SLC22A2 and RASSF1A in HCC and adjacent non-tumor tissue. Figure S5: association between SLC22A1 mRNA expression or DNA methylation and tumor stage or histological tumor grade. Figure S6: DNA methylation profiles of SLC22A1, SLC22A2, and SLC22A3 in non-tumor (normal) liver tissues, as well as in non-tumor liver tissue derived from patients with HCC. [file gm298-S1.PDF]

Additional file 1:

Schaeffeler *et al.:* DNA methylation is associated with downregulation of the organic cation transporter OCT1 (SLC22A1) in human hepatocellular carcinoma

Methods: **page 2-4**

Tables S1 to S5: **page 5-9**

Figures S1 to S6: **page 10-15**

Methods

TaqMan analysis

mRNA expression was quantified by TaqMan technology using a 7900HT Real-Time PCR System (Applied Biosystems, Foster City, CA, USA) and a BioMark^{TM} System (Fluidigm, South San Francisco, CA, USA). Expression levels of pooled cDNA samples were quantified using predeveloped mRNA expression assays (Applied Biosystems, Foster City, CA, USA) and relative expression was calculated using Δ Ct-method. Expression levels in cDNA samples from 381 human tissues, using commercial arrays, were determined as previously described [20]. For analysis of gene expression on the BioMark^{TM} System, 1.25 µl of reverse transcribed cDNA (corresponding to 31.25 ng RNA) from each sample was preamplified by using the TaqMan PreAmp Master Mix Kit (Applied Biosystems). The samples were preamplified for 14 cycles and then diluted 1:5 with DNA suspension buffer (TEKnova, USA). Samples and predeveloped assays (Applied Biosystems, Foster City, CA, USA) were loaded on a BioMark^{TM} 48.48 Dynamic Array. 2.25 µl of the preamplified cDNA together with 2.5 µl of 2x Master Mix (Applied Biosystems) and 0.25 µl Fluidigm Loading Agent was loaded into the Dynamic Array on the IFC controller (Fluidigm, South San Francisco, CA, USA). Data was collected and analysed on the BioMark Instrument. Each sample was setup in triplicate. Level of expression of each mRNA was normalized to the level of β-actin mRNA.

Immunofluorescence microscopy of tissue samples

Antibodies

The polyclonal antisera KEN, KEK, and CGR were raised in rabbits against synthetic peptides corresponding to the carboxy-terminal sequences of human OCT1, OCT2, and OCT3, respectively, as described [20,45]. The mouse monoclonal antibody against dipeptidylpeptidase IV (DPPIV, CD26) was from Ancell Corp. (Bayport, MN, USA). Alexa Fluor488-conjugated goat anti-rabbit IgG and Alexa Fluor546-conjugated goat anti-mouse IgG were from Invitrogen (Carlsbad, CA).

Immunofluorescence

Cryosections of human tissue samples (5-7 µm) were prepared and fixed with methanol as described [20,45]. Cryosections were incubated with the primary antibody and subsequently the corresponding secondary antibody for 1 hour. Antibodies were diluted

in PBS as follows: KEK and CGR 1:100, KEN and fluorochrome-conjugated secondary antibodies 1:300, anti-CD26 1:400. Images were taken with a confocal laser scanning microscope (TCS NT Confocal System, Leica Microsystems, Wetzlar, Germany).

DNA methylation analysis

Genomic DNA from tissue samples was extracted using the QIAmp DNA Mini Kit (Qiagen, Hilden, Germany) following the manufacturer's instructions. Bisulfite treatment was performed by using the EZ DNA Methylation-Gold™ Kit (Zymo Research Corporation, Orange, CA, USA). Genomic DNA was converted as described in the manufacturer's instructions.

Quantitative DNA methylation analysis was performed with MALDI-TOF mass spectrometry (Sequenom, San Diego, CA, USA). Generally, the overall process variability of bisulfite-based PCR methods, like MALDI-TOF MS, results in standard deviations of 5% to 8%.The Sequenom EpiTyper technology has a detection limit of ~5% methylated DNA and the accuracy and robustness for quantification of DNA methylation has been demonstrated previously [24,47]. All PCR primers were designed with Methprimer software [46]. For *SLC22A1* and *SLC22A3* amplicons were designed that cover the promoter region, as well as a predicted CpG island [46] in the 5`UTR region of *SLC22A3*. The promoter region was therefore divided into three regions and amplified by three different primer sets for *SLC22A1* and *SLC22A3*. Fragment SLC22A3_06 was designed according to Ehrich et al. 2008. For *SLC22A2* amplicons were designed in the 5` UTR region, previously identified by Aoki et al. [16]. DNA methylation of *RASSF1A* was analysed as described previously [47].

An additional T7 promoter tag was added to each reverse primer, as well as an additional 10-mer tag on the forward primer. All primer sets are available upon request. The T7 recognition sequence allows the generation of a single stranded RNA copy of the template, which can then be cleaved base specifically. MassCLEAVE reactions were performed as described previously [23,24]. Mass spectra were acquired with the MassArray Compact MALDI-TOF MS (Sequenom) and spectra methylation ratios were analysed by using the Epityper 1.0 software. The MassCLEAVE MALDI-TOF mass spectrometry method does not always allow detection of methylation levels for each single CpG site. Therefore some of the methylation values represent the methylation state of subsequent CpG sites (see additional file 1, table S5). Moreover some CpG sites will be missed because masses of the respective fragments are outside the used mass range (~1700-7000Da). Additionally some CpG sites could not be reliably quantified

because of overlapping fragments in the mass spectrum. In total, 18 CpG sites were quantified for *SLC22A1*, 6 sites for *SLC22A2* and 57 sites for *SLC22A3* (marked in black in additional file 1, table S5).

Statistical methods

Statistical analyses were performed using the statistical software R, version 2.13.0 [48], PASW Statistics 17.0.2 and GraphPadPrism 4.03. Unpaired Wilcoxon-Mann-Whitney-tests were applied to compare (a) methylation values of individual CpG sites between different tissue types, as well as (b) mRNA or protein expression in HCC and paired adjacent non-tumor tissue. In HCC tumor samples (n=22), associations between *SLC22A1* DNA methylation of individual CpG sites and SLC22A1 mRNA expression were analyzed using two-sided Spearman`s correlation tests. Associations between score intensities, mRNA or DNA methylation levels measured in non-tumor and HCC tissues and clinicopathological variables (e.g. age, sex, MIB1/Ki-67, etiology, tumor grade and stage) were analyzed by unpaired Wilcoxon-Mann-Whitney-Tests, Kruskal-Wallis Test and Spearman's correlation tests as appropriate. Wilcoxon signed rank tests were used to investigate differences in score intensities between pairs of non-tumor and HCC tissues.

R-package e1071 (version 1.5-26) was applied to classify HCC and normal tissues by *SLC22A1* and *RASSF1A* methylation values, using support vector machines with RBF kernel. Initial feature selection was based on results of univariate analysis, choosing those CpG sites with unadjusted $P < 0.001$ in unpaired Wilcoxon-Mann-Whitney tests and a difference in median methylation levels > 20% between HCC and normal tissues. Class weights for classification were chosen proportional to reciprocals of numbers of HCC (n=22) and normal tissues (n=100). 10-fold cross validation was used to determine choice of parameter gamma of RBF kernel.

Where indicated, p-values were adjusted according to Holm`s multiple testing correction procedure [49]. Statistical significance was defined as $P<0.05$.

Table S1. Overview about different sample sets used in the present study

Sample set	Description (origin)	Application
TissueScan	n=381 cDNA samples from 20 different tissues (OriGene, Rockville, MD, USA)	used for mRNA analysis
HCC study population (set1) (detailed description see table S2)	n=71 paraffin-embedded tissue samples from hepatocellular carcinoma and corresponding adjacent non-tumor tissue (University Hospital Regensburg, Regensburg, Germany)	used for semiquantitative protein expression analysis on TMAs
HCC study population (set2) (detailed description see table S3)	n=7 (cohort 1) fresh-frozen HCC tissues (OriGene, Rockville, MD, USA)<br>n=15 (cohort 2) fresh-frozen HCC tissue and adjacent normal tissue (University Hospital Regensburg, Regensburg, Germany)	used for mRNA as well as DNA methylation analysis
Liver tissue collection (IKP-liverbank) (detailed description see table S4)	n=100 histologically normal liver samples derived from patients without HCC	used for mRNA and DNA methylation analysis
	n=20 histologically normal liver samples derived from patients with HCC	used for DNA methylation analysis

Table S2. Clinicopathological characteristics of 71 patients with hepatocellular carcinoma

Variable	Category	n (% of total)
Age at surgery	< 60 years	33 (46.5%)
	≥ 60 years	38 (53.5%)
Sex	male	57 (80.3%)
	female	14 (19.7%)
T-stage	pT1	15 (21.1%)
	pT2	23 (32.4%)
	pT3	31 (43.7%)
	pT4	2 (2.8%)
Histological grade	G1	24 (33.8%)
	G2	39 (54.9%)
	G3	8 (11.3%)
Etiology	Viral (HBV/HCV)	11 (15.5%)
	Alcohol	29 (40.8%)
	Viral and alcohol	3 (4.2%)
	cryptogenic and others	20 (28.2%)
	no liver disease	8 (11.3%)
Proliferation rate<br>Ki-67/MIB1 (mean±SD)		15.6±21.4

Table S3. Clinicopathological characteristics of HCC study population

		Cohort 1 (n=7)	Cohort 2 (n=15)
Tumor Stage			
	T1	1	6
	T2	2	3
	T3	4	4
	T4	-	1
	nd	-	1
Histological Grade			
	1	4	3
	2	3	8
	3	-	3
	nd	-	1
Age at surgery			
	< 60 years	1	6
	≥ 60 years	6	9
Sex			
	male	5	14
	female	2	1
Size			
	< 5cm	2	6
	≥ 5cm	5	7
	nd	-	2

Table S4. Characteristics of histologically normal liver tissues (IKP-liverbank)

Indication of hepatectomy	n
Liver metastasis (e.g. after colon cancer)	81
Haemangioma	8
Caroli syndrome	3
Liver cyst	2
Other	6
**Total**	**100**
Hepatocellular carcinoma	20
**Total**	**120**

Liver tissue samples were collected from patients undergoing liver surgery ("indication of hepatectomy") at the Department of General, Visceral and Transplantation Surgery (A.K.Nuessler, P.Neuhaus, Campus Virchow, University Medical Center Charité, Humboldt University Berlin, Germany). As previously described in detail (Nies et al. 2009), all liver tissue samples were examined by a pathologist and only histologically normal liver tissue was used for this study.

Table S5. DNA methylation analysis of *OCT1/SLC22A1*, *OCT2/SLC22A2* and *OCT3/SLC22A3*.

SLC22A1	
Position	CpG site
-426	SLC22A1_03_CpG_1
-309	SLC22A1_03_CpG_2
-257	SLC22A1_03_CpG_3
-236 / -230	SLC22A1_03_CpG_4.5
-217	SLC22A1_03_CpG_6
-195	SLC22A1_03_CpG_7
-24	SLC22A1_04_CpG_1
+9	SLC22A1_04_CpG_2
+86 / +98	SLC22A1_04_CpG_3.4
+117	SLC22A1_04_CpG_5
+181	SLC22A1_04_CpG_6
+200	SLC22A1_04_CpG_7
+237 / +239 / +243	SLC22A1_04_CpG_8.9.10
+268	SLC22A1_04_CpG_11
+294	SLC22A1_02_CpG_1
+350	SLC22A1_02_CpG_2
+386 / +390	SLC22A1_02_CpG_3.4
+402	SLC22A1_02_CpG_5

SLC22A2	
Position	CpG site
-527	SLC22A2_01_CpG_1
-503	SLC22A2_01_CpG_2
-484	SLC22A2_01_CpG_3
-462	SLC22A2_01_CpG_4
-425 / -420	SLC22A2_01_CpG_5.6
-384	SLC22A2_01_CpG_7
-374	SLC22A2_01_CpG_8

SLC22A3	
Position	CpG site
-1037 / -1030	SLC22A3_01_CpG_1.2
-1024 / -1016	SLC22A3_01_CpG_3.4
-1005 / -1003	SLC22A3_01_CpG_5.6
-993	SLC22A3_01_CpG_7
-984	SLC22A3_01_CpG_8
-947	SLC22A3_01_CpG_9
-933 / -931 / -923	SLC22A3_01_CpG_10.11.12
-911	SLC22A3_01_CpG_13
-898	SLC22A3_01_CpG_14
-854	SLC22A3_01_CpG_15
-845	SLC22A3_01_CpG_16
-821	SLC22A3_01_CpG_17
-574	SLC22A3_06_CpG_1
-561	SLC22A3_06_CpG_2
-557 / -555	SLC22A3_06_CpG_3.4
-524	SLC22A3_06_CpG_5
-511	SLC22A3_06_CpG_6
-505 / -500 / -498 / -496 / -494 / -492 / -490	SLC22A3_06_CpG_7.8.9.10.11.12.13
-477	SLC22A3_06_CpG_14
-465 / -460	SLC22A3_06_CpG_15.16
-449	SLC22A3_06_CpG_17
-433	SLC22A3_06_CpG_18
-423	SLC22A3_06_CpG_19
-390	SLC22A3_06_CpG_20
-338 / -336	SLC22A3_07_CpG_1.2
-328	SLC22A3_07_CpG_3
-319 / -315	SLC22A3_07_CpG_4.5
-308 / -306 / -300 / -296 / -285 / -283	SLC22A3_07_CpG_6.7.8.9.10.11
-272	SLC22A3_07_CpG_12
-267 / -263	SLC22A3_07_CpG_13.14
-253 / -251 / -249	SLC22A3_07_CpG_15.16.17
-240/-236/-230/-227/-221/-219/-215/-206/-204	SLC22A3_07_CpG_18.19.20.21.22.23.24.25.26
-192 / -181 / -177 / -173	SLC22A3_07_CpG_27.28.29.30
-160 / -153 / -151	SLC22A3_07_CpG_31.32.33
-135 / -129 / -125	SLC22A3_07_CpG_34.35.26
-117	SLC22A3_07_CpG_37
-109	SLC22A3_07_CpG_38
-100 / -93 / -91	SLC22A3_07_CpG_39.40.41
-82 / -79	SLC22A3_07_CpG_42.43
-63 / -60 / -50 / -48	SLC22A3_07_CpG_44.45.46.47
-38	SLC22A3_07_CpG_48
-33 / -31 / -24 / -20 / -17 / -13 / -10 / -6	SLC22A3_07_CpG_49.50.51.52.53.54.55.56
+12	SLC22A3_07_CpG_57
+15	SLC22A3_07_CpG_58
+20	SLC22A3_07_CpG_59
+28 / +36	SLC22A3_07_CpG_60.61
+42 / +46	SLC22A3_07_CpG_62.63
+58 / +60	SLC22A3_07_CpG_64.65

CpG sites marked in black are quantified using MassCLEAVE MALDI-TOF mass spectrometry. Some of the methylation values represent the methylation state of subsequent CpG sites. Positions are given relative to respective ATG start site.

Figure S1. Differences in the SLC22A1 immunohistochemical score between the corresponding adjacent non-tumor tissue and HCC tissue, regarding the underlying etiology for HCC

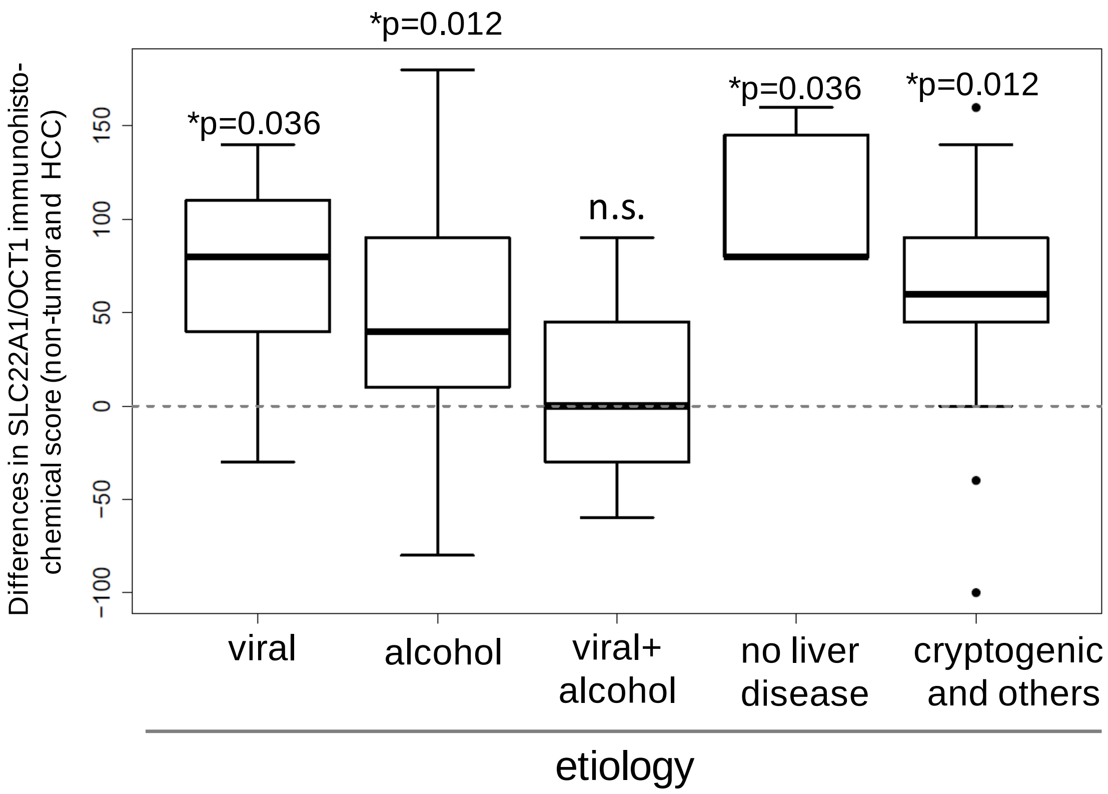

Figure S1: Significant differences, even after correction for multiple testing, were observed for all groups except for patients with viral and alcohol etiology, which may be due to the small sample size in this group (n=3, see additional file 1, table S2). Wilcoxon signed rank tests were used to investigate differences in score values between pairs of non-tumor and HCC tissues.*Holm-adjusted p-values are given.

Figure S2.

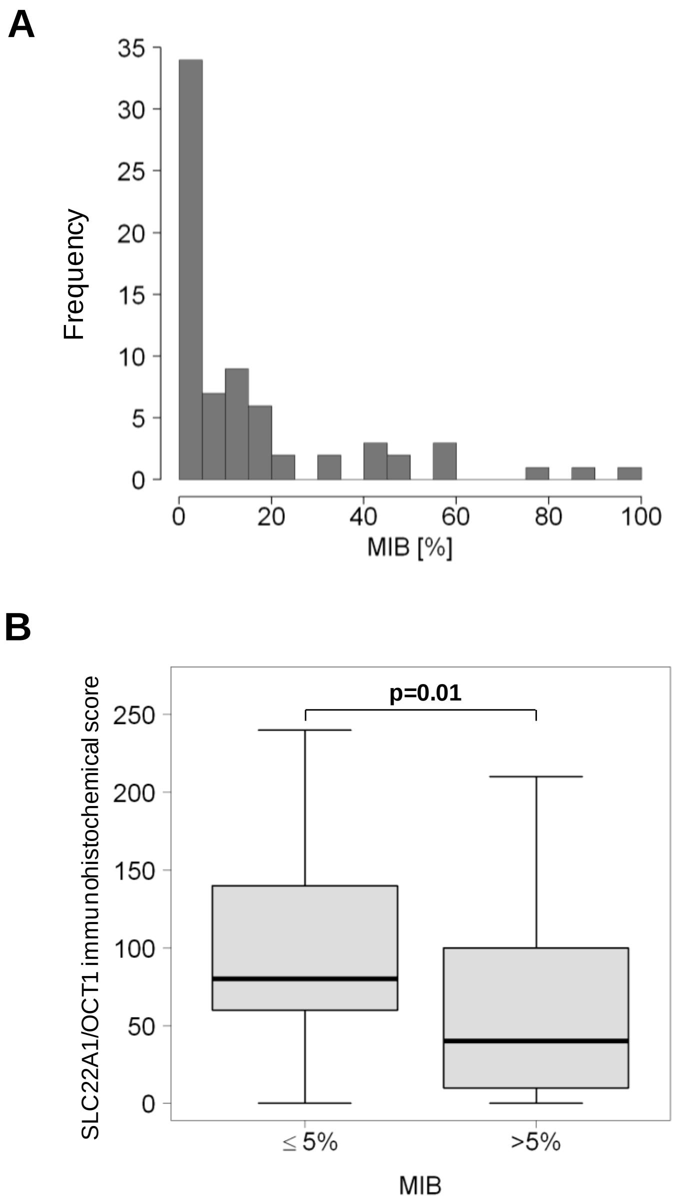

Figure S2: (A) Histogram of Ki-67/MIB1 data in HCC tumor samples. Ki-67/MIB1 was analysed by semiquantitative immunohistochemistry. (B) A significant difference in SLC22A1 expression between samples with low (≤5%) and high (>5%) proliferation rate was found (p=0.01).

Figure S3.

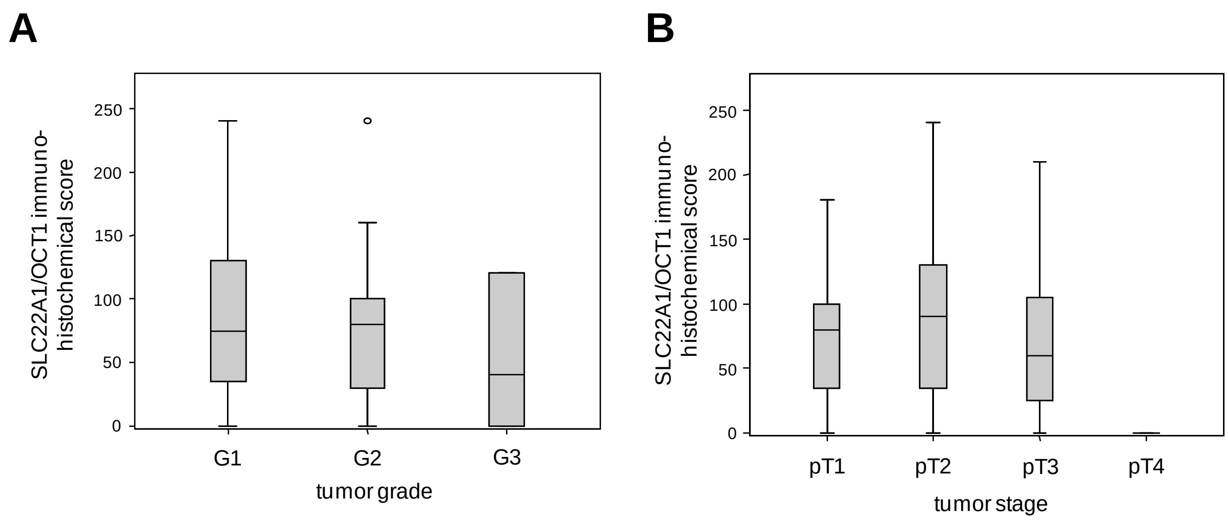

Figure S3: Association between SLC22A1 protein expression (determined by immunohistochemistry) and tumor grade (A) or tumor stage (B).

Figure S4.

A

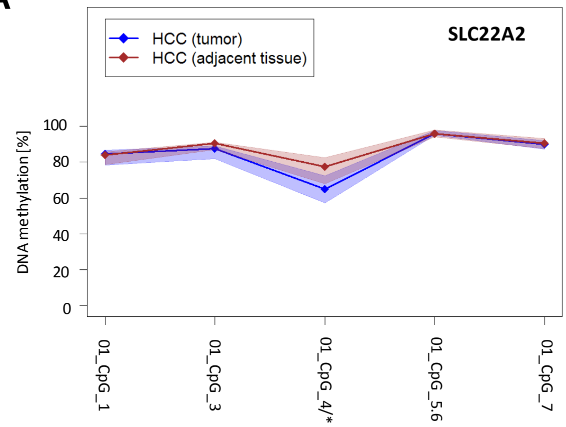

B

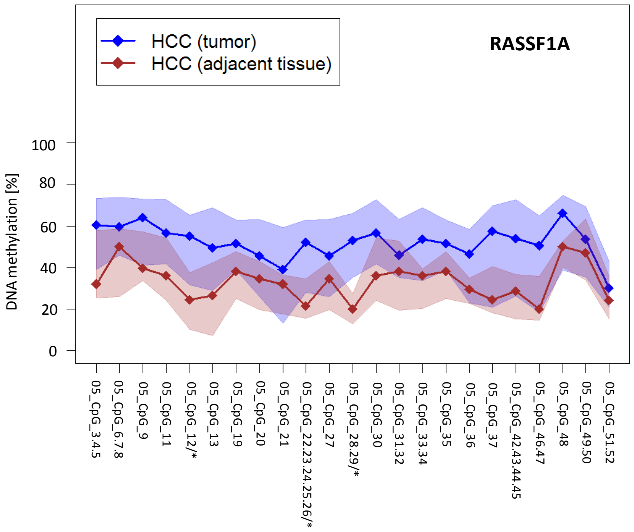

Figure S4: DNA methylation profiles of *SLC22A2* (A) and *RASSF1A* (B) in HCC (n=22) and adjacent non-tumor tissue. DNA methylation levels (y-axis) for each individual CpG site (x-axis) are given. Methylation profiles are showing median methylation levels (diamonds) at each CpG position (shaded areas are defined by 25%/75% quantiles). Differences in methylation levels were tested for each CpG site. *p<0.05, **p<0.01.

Figure S5.

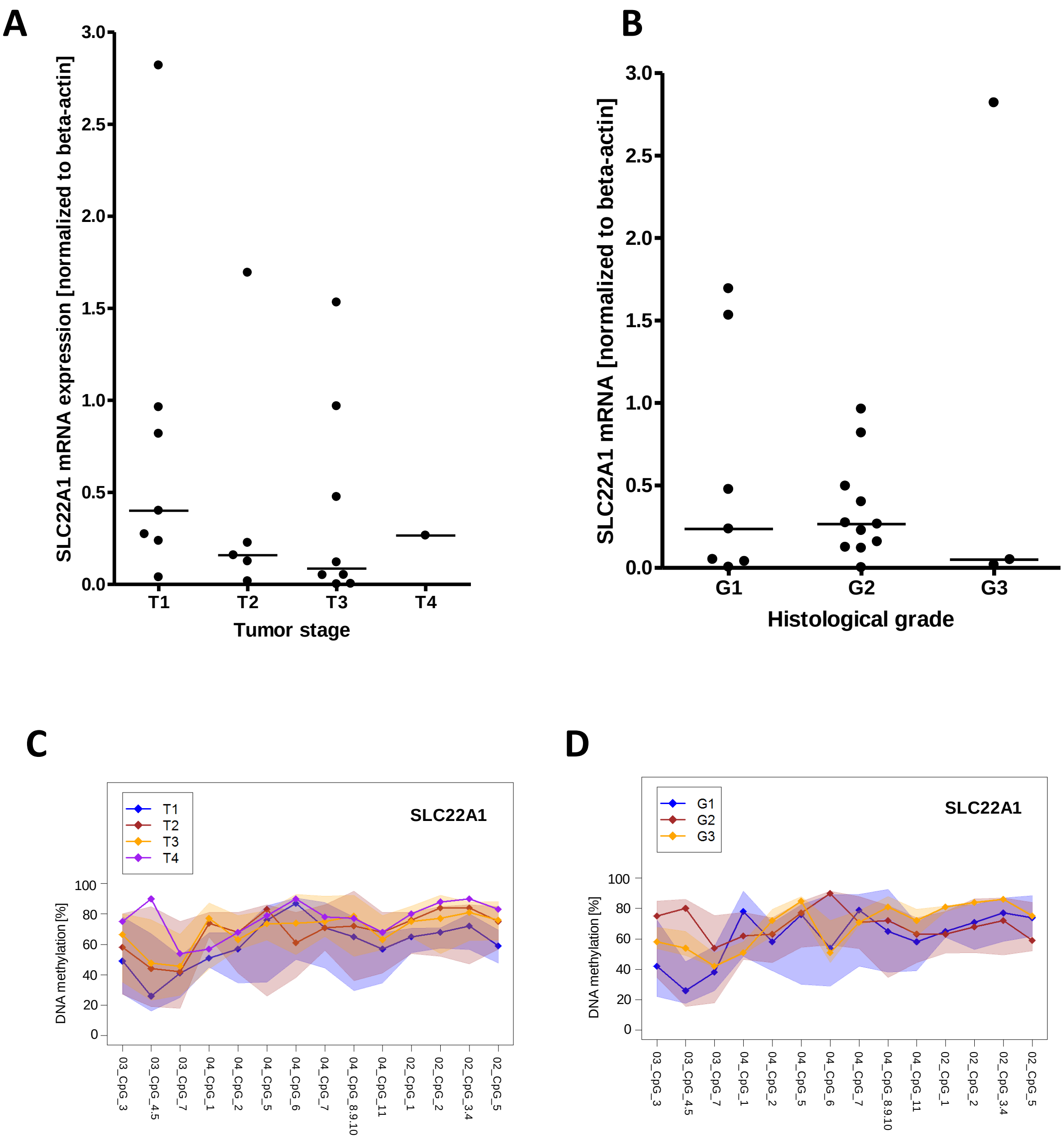

Figure S5: Association between SLC22A1 mRNA expression and tumor stage (A) or histological tumor grade (B). Relationship between *SLC22A1* DNA methylation and tumor stage (C) or tumor grade (D).

Figure S6.

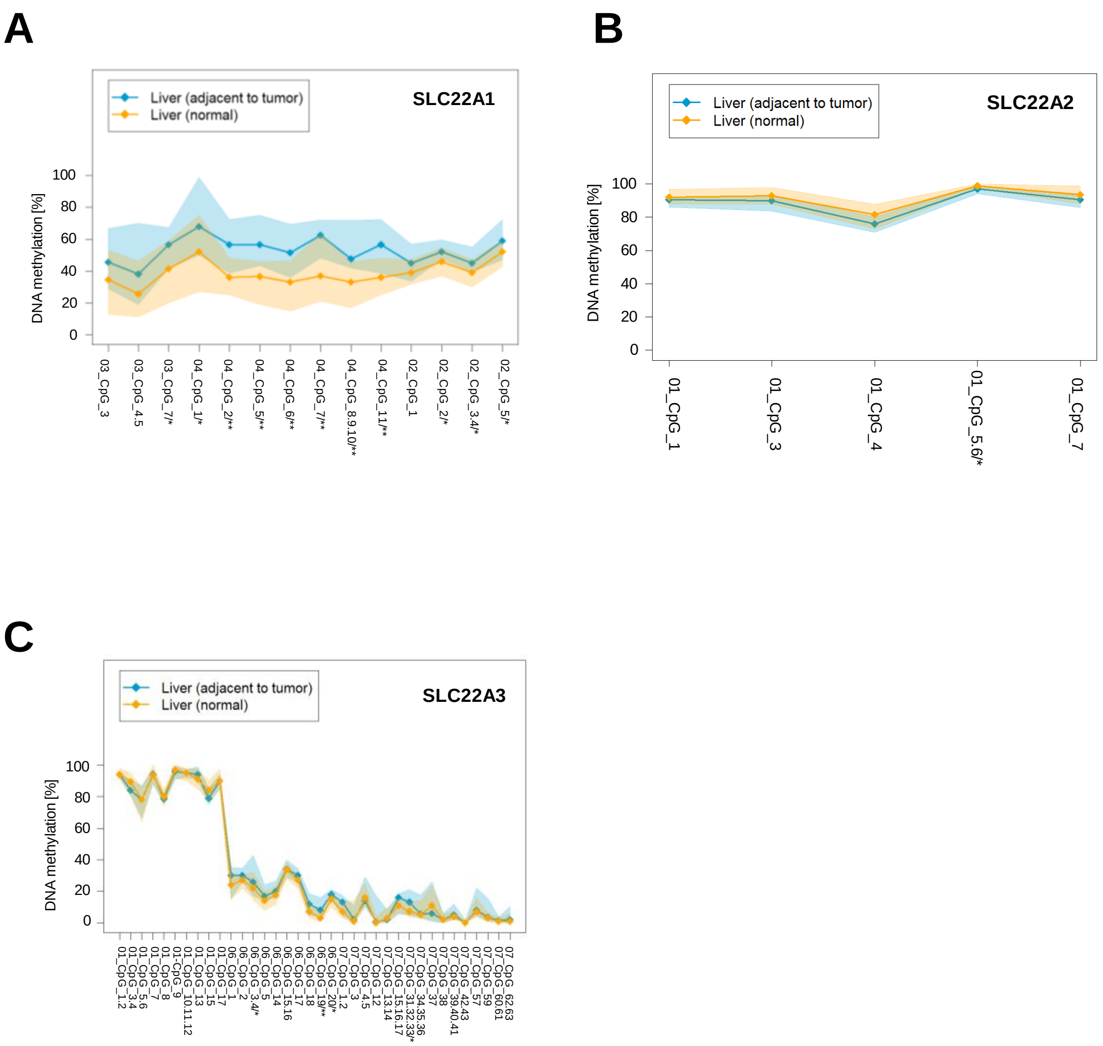

Figure S6. DNA methylation profiles of *SLC22A1* (A), *SLC22A2* (B), and *SLC22A3* (C) in non-tumor (normal) liver tissues (n=100, IKP-liverbank), as well as in non-tumor liver tissue derived from patients with hepatocellular carcinoma (n=20). Differences in methylation level were tested for each CpG site. p-values *p<0.05, **p<0.01. DNA methylation levels (y-axis) for each individual CpG site (x-axis) is given. Methylation profiles are showing median methylation levels (diamonds) at each CpG position (shaded areas are defined by 25%/75% quantiles).
